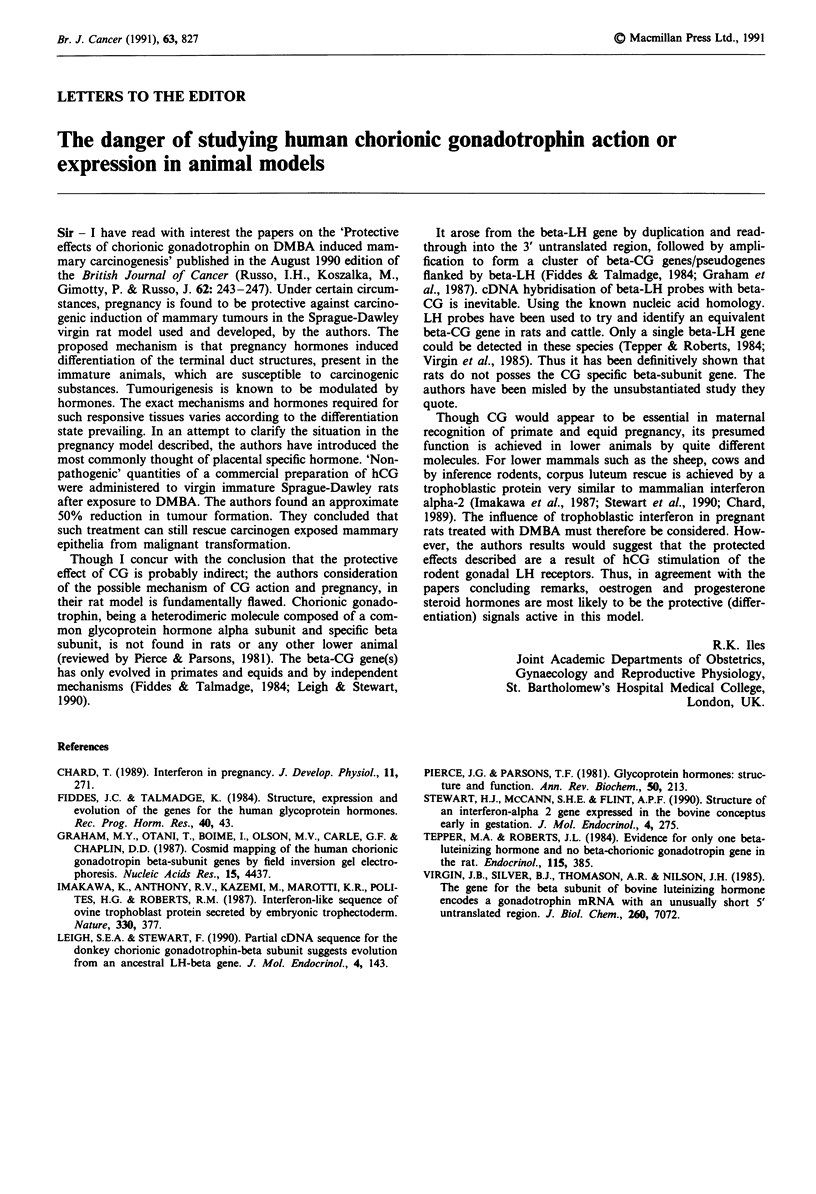# The danger of studying human chorionic gonadotrophin action or expression in animal models.

**DOI:** 10.1038/bjc.1991.184

**Published:** 1991-05

**Authors:** R. K. Iles


 © Macmillan Press Ltd., 1991

### LETTERS TO THE EDITOR

### The danger of studying human chorionic gonadotrophin action or expression in animal models

**Sir** – I have read with interest the papers on the 'Protective effects of chorionic gonadotrophin on DMBA induced mammary carcinogenesis' published in the August 1990 edition of the *British Journal of Cancer* (Russo, I.H., Koszalka, M., Gimotty, P. & Russo, J. **62:** 243–247). Under certain circumstances, pregnancy is found to be protective against carcinogenic induction of mammary tumours in the Sprague-Dawley virgin rat model used and developed, by the authors. The proposed mechanism is that pregnancy hormones induced differentiation of the terminal duct structures, present in the immature animals, which are susceptible to carcinogenic substances. Tumourigenesis is known to be modulated by hormones. The exact mechanisms and hormones required for such responsive tissues varies according to the differentiation state prevailing. In an attempt to clarify the situation in the pregnancy model described, the authors have introduced the most commonly thought of placental specific hormone. 'Non-pathogenic' quantities of a commercial preparation of hCG were administered to virgin immature Sprague-Dawley rats after exposure to DMBA. The authors found an approximate 50% reduction in tumour formation. They concluded that such treatment can still rescue carcinogen exposed mammary epithelia from malignant transformation.

Though I concur with the conclusion that the protective effect of CG is probably indirect; the authors consideration of the possible mechanism of CG action and pregnancy, in their rat model is fundamentally flawed. Chorionic gonadotrophin, being a heterodimeric molecule composed of a common glycoprotein hormone alpha subunit and specific beta subunit, is not found in rats or any other lower animal (reviewed by Pierce & Parsons, 1981). The beta-CG gene(s) has only evolved in primates and equids and by independent mechanisms (Fiddes & Talmadge, 1984; Leigh & Stewart, 1990). It arose from the beta-LH gene by duplication and read-through into the 3′ untranslated region, followed by amplification to form a cluster of beta-CG genes/pseudogenes flanked by beta-LH (Fiddes & Talmadge, 1984; Graham *et al.*, 1987). cDNA hybridisation of beta-LH probes with beta-CG is inevitable. Using the known nucleic acid homology. LH probes have been used to try and identify an equivalent beta-CG gene in rats and cattle. Only a single beta-LH gene could be detected in these species (Tepper & Roberts, 1984; Virgin *et al.*, 1985). Thus it has been definitively shown that rats do not posses the CG specific beta-subunit gene. The authors have been misled by the unsubstantiated study they quote.

Though CG would appear to be essential in maternal recognition of primate and equid pregnancy, its presumed function is achieved in lower animals by quite different molecules. For lower mammals such as the sheep, cows and by inference rodents, corpus luteum rescue is achieved by a trophoblastic protein very similar to mammalian interferon alpha-2 (Imakawa *et al.*, 1987; Stewart *et al.*, 1990; Chard, 1989). The influence of trophoblastic interferon in pregnant rats treated with DMBA must therefore be considered. However, the authors results would suggest that the protected effects described are a result of hCG stimulation of the rodent gonadal LH receptors. Thus, in agreement with the papers concluding remarks, oestrogen and progesterone steroid hormones are most likely to be the protective (differentiation) signals active in this model.

R.K. Iles
Joint Academic Departments of Obstetrics,
Gynaecology and Reproductive Physiology,
St. Bartholomew's Hospital Medical College,
London, UK.

### References

CHARD, T. (1989). Interferon in pregnancy. *J. Develop. Physiol.*, **11**, 271.

FIDDES, J.C. & TALMADGE, K. (1984). Structure, expression and evolution of the genes for the human glycoprotein hormones. *Rec. Prog. Horm. Res.*, **40**, 43.

GRAHAM, M.Y., OTANI, T., BOIME, I., OLSON, M.V., CARLE, G.F. & CHAPLIN, D.D. (1987). Cosmid mapping of the human chorionic gonadotropin beta-subunit genes by field inversion gel electrophoresis. *Nucleic Acids Res.*, **15**, 4437.

IMAKAWA, K., ANTHONY, R.V., KAZEMI, M., MAROTTI, K.R., POLITES, H.G. & ROBERTS, R.M. (1987). Interferon-like sequence of ovine trophoblast protein secreted by embryonic trophectoderm. *Nature*, **330**, 377.

LEIGH, S.E.A. & STEWART, F. (1990). Partial cDNA sequence for the donkey chorionic gonadotrophin-beta subunit suggests evolution from an ancestral LH-beta gene. *J. Mol. Endocrinol.*, **4**, 143.

PIERCE, J.G. & PARSONS, T.F. (1981). Glycoprotein hormones: structure and function. *Ann. Rev. Biochem.*, **50**, 213.

STEWART, H.J., McCANN, S.H.E. & FLINT, A.P.F. (1990). Structure of an interferon-alpha 2 gene expressed in the bovine conceptus early in gestation. *J. Mol. Endocrinol.*, **4**, 275.

TEPPER, M.A. & ROBERTS, J.L. (1984). Evidence for only one beta-luteinizing hormone and no beta-chorionic gonadotropin gene in the rat. *Endocrinol.*, **115**, 385.

VIRGIN, J.B., SILVER, B.J., THOMASON, A.R. & NILSON, J.H. (1985). The gene for the beta subunit of bovine luteinizing hormone encodes a gonadotrophin mRNA with an unusually short 5′ untranslated region. *J. Biol. Chem.*, **260**, 7072.